# Faecal contamination of the environment and child health: a systematic review and individual participant data meta-analysis

**DOI:** 10.1016/S2542-5196(20)30195-9

**Published:** 2020-09-09

**Authors:** Frederick G B Goddard, Amy J Pickering, Ayse Ercumen, Joe Brown, Howard H Chang, Thomas Clasen

**Affiliations:** a Gangarosa Department of Environmental Health, Rollins School of Public Health, Emory University, Atlanta, GA, USA; b Department of Biostatistics and Bioinformatics, Rollins School of Public Health, Emory University, Atlanta, GA, USA; c Department of Epidemiology, Harvard TH Chan School of Public Health, Boston, MA, USA; d Department of Civil and Environmental Engineering, School of Engineering, Tufts University, Medford, MA, USA; e Department of Forestry and Environmental Resources, North Carolina State University, Raleigh, NC, USA; f School of Civil and Environmental Engineering, Georgia Institute of Technology, Atlanta, GA, USA

## Abstract

**Background:**

Exposure to faecal contamination is believed to be associated with child diarrhoea and possibly stunting; however, few studies have explicitly measured the association between faecal contamination and health. We aimed to assess individual participant data (IPD) across multiple trials and observational studies to quantify the relationship for common faecal–oral transmission pathways.

**Methods:**

We did a systematic review and meta-analysis of IPD from studies identified in an electronic search of PubMed, Web of Science, and Embase on May 21, 2018. The search was done in English, but full texts published in French, Portuguese, and Spanish were also reviewed. Eligible studies quantified (1) household-level faecal indicator bacteria concentrations along common faecal-oral transmission pathways of drinking water, soil, or food, on children's hands or fomites, or fly densities in food preparation areas; and (2) individual-level diarrhoea or linear growth measures for children younger than 5 years in low-income and middle-income countries. For the diarrhoea analysis, all definitions of diarrhoea were eligible but studies were excluded if they used a recall period longer than 7 days. For the linear growth analysis (using height-for-age Z scores [HAZ]), cross-sectional studies were excluded, because of the absence of longitudinal environmental contamination data measured before the growth outcomes. We used multilevel generalised mixed-effects models to estimate the odds ratio (OR) for diarrhoea and the difference in HAZ scores for individual studies associated with a 1-log_10_ higher measure of faecal contamination. Estimates from each study were combined under a random-effects meta-analysis framework. The study protocol was pre-registered with PROSPERO (CRD42018102114).

**Findings:**

From 72 eligible studies, we included IPD for 20 studies in the meta-analyses, totalling 54 225 diarrhoea or linear growth observations matched to faecal indicator bacteria concentrations in drinking water, and a further 35 010 observations with faecal contamination data for the other transmission pathways. Child diarrhoea was associated with 1-log_10_ higher faecal indicator bacteria concentrations in drinking water (OR 1·09, 95% CI 1·04 to 1·13; p=0·0002, *I*^2^=34%, 95% CI 0 to 62) and on children's hands (1·11, 1·02 to 1·22; p=0·021, *I*^2^=0%, 0 to 71). Lower HAZ scores were associated with 1-log_10_ higher median faecal indicator bacteria concentrations in drinking water (HAZ −0·04, 95% CI −0·06 to −0·01; p=0·0054; *I*^2^=19%, 95% CI 0 to 63) and on fomites (–0·06, −0·12 to 0·00; p=0·044, *I*^2^=57%, 0 to 90).

**Interpretation:**

Although summary measures from individual studies often report little or no effect of measured faecal contamination on child health, this multi-study IPD analysis indicates that household faecal indicator bacteria concentrations are associated with important adverse health outcomes in young children. Improved direct measures of exposure and enteric pathogens could help to better characterise the relationship and inform intervention design in future studies.

**Funding:**

None.

## Introduction

Globally, diarrhoea represents the fourth leading cause of death among children under 5 years of age, accounting for approximately 534 000 deaths in 2017.^1^ An estimated 151 million children were stunted in 2018 (stunting being defined as a height-for-age Z-score more than two SDs below the WHO child growth standards reference median for age and sex). 91% of these stunted children were living in low-income or lower-middle-income countries,^2^ and the burden of diarrhoeal diseases and nutritional deficits is most pronounced in these countries.^3^ An estimated 62% of deaths from diarrhoea and 16% of cases of malnutrition among children younger than 5 years are linked to faecal exposure from poor drinking water, sanitation, and hygiene (WaSH) behaviours.^4^

Traditional WaSH approaches have focused on reducing open defecation, promoting improved sanitation, encouraging handwashing with soap, and improving the quality of and access to water. Systematic reviews of these interventions have found them to be protective against diarrhoea and malnutrition; however, the evidence is mostly considered to be of low quality.^5^, ^6^, ^7^ Rigorous evaluations of WaSH interventions have reported mixed results, finding either no evidence of health benefits,^8^, ^9^, ^10^ a reduction in diarrhoea but no improvement in child growth,^11^ or improved growth but no effect on diarrhoea.^12^, ^13^ The theory of change underlying WaSH interventions is that they prevent disease by interrupting the common faecal-oral transmission pathways: hands, flies, food, drinking water, soil, and fomites.^14^, ^15^ The mixed effect of WaSH interventions on child health outcomes could indicate that either the interventions did not sufficiently reduce exposure to faecal contamination and subsequent enteric infections,^16^, ^17^ or that household faecal contamination is not a major cause of child diarrhoea and growth faltering.

**Research in context**

**Evidence before this study**

Although reducing faecal contamination in the household environment is an essential condition to improving health from water, sanitation and hygiene (WaSH) interventions, few studies actually measure the association between faecal contamination and health or the effects of interventions on faecal exposure. Those that do are largely confined to measurements of drinking water quality, one of multiple sources of exposure including food and contact with contaminated surfaces. Pooled analyses of these studies were limited to faecal contamination in drinking water and diarrhoea, and yielded differing results. A study by Gundry and colleagues reported no association between three types of faecal indicator bacteria and diarrhoea (odds ratio [OR] 1·12, 95% CI 0·85–1·48); another by Gruber and colleagues found a significant association between concentrations of *Escherichia coli* in drinking water and diarrhoea (relative risk 1·54, 95% CI 1·37–1·74) but not for faecal coliforms (1·07, 0·79–1·45); and a third by Hodge and colleagues showed a higher risk of diarrhoea with higher concentrations of faecal coliforms in drinking water (OR 1·18, 95% CI 1·11–1·26). A study by Pickering and colleagues in rural Bangladesh investigated the relationship between household faecal contamination along multiple pathways (water, soil, food, hands, and flies) and WHO-defined and caregiver-defined diarrhoea, and bloody stool, measured concurrently and prospectively. Results from this study were mixed, finding evidence of an association between *E coli* on children's hands and WHO-defined and caregiver defined diarrhoea (incident rate ratio [IRR] 1·23, 95% CI 1·06–1·43; and 1·31, 1·11–1·55, respectively) and between *E coli* in food and bloody stool (IRR 1·34, 95% CI 1·07–1·68) when samples were collected prospectively. This study also found an association between *E coli* in flies and soil and caregiver-defined diarrhoea (prevalence ratio 1·15, 95% CI 1·04–1·26; and 1·16, 1·02–1·32, respectively) when samples were collected concurrently with diarrhoea data.

**Added value of this study**

Our analysis of nearly 90 000 individual participant data points from 20 studies across a range of low-income and middle-income settings provides evidence of an association between contaminated drinking water and both diarrhoea and impaired linear growth in children. It also implicates contaminated children's hands in diarrhoea and fomites in linear growth. The study highlights the paucity of evidence on other probable exposure pathways, especially food and soil.

**Implications of all the available evidence**

Despite the uncertainty from individual studies and previous reviews, our findings provide evidence of the fundamental association between faecal contamination at the household level and diarrhoea, still a major killer of young children. The study also delivers evidence on the effects of faecal contamination on impaired linear growth. These results suggest that the failure of WaSH interventions to achieve consistent improvements in health might be due to their failure to adequately reduce faecal contamination. The study also indicates the need for more comprehensive and rigorous improvements in exposure assessments in WaSH research.

Except for drinking water, however, few studies that assess the effect of interventions on health actually measure the effects on household faecal contamination and those that do often report little effect on other transmission pathways.^18^ Previous reviews that examined the relationship between indicators of faecal contamination and health were limited to drinking water and focused solely on diarrhoeal disease. Two systematic reviews using aggregate data reported conflicting results, one finding no association between three types of faecal indicator bacteria and diarrhoea and the other showing an association between concentrations of *Escherichia coli* in drinking water and diarrhoea.^19^, ^20^ A study using individual participant data (IPD) reported an association between faecal coliforms in drinking water and diarrhoea,^21^ but only included seven studies. Mixed results from these studies and from WaSH evaluations have raised questions about the relative contribution of different faecal-oral transmission pathways to adverse acute health outcomes, and the relationship between faecal contamination and child growth.

Therefore, our aim was to do a systematic review and meta-analysis using IPD to examine the relationship between faecal contamination and child health. Specifically, we sought to test whether faecal contamination along transmission pathways, measured using faecal indicator bacteria and fly densities, was associated with diarrhoea and linear growth in children under age 5 years in low-income and middle-income countries. Unlike conventional meta-analyses that pool available estimates of effect from eligible studies, the use of IPD enables consistent analytical approaches across individual studies and assessment of the relationships between child health and faecal contamination along common exposure pathways, even when the individual studies did not publish the effect estimates of interest.^22^

## Methods

### Search strategy and selection criteria

We did a systematic review and meta-analysis of IPD from studies identified in an electronic search of the PubMed, Web of Science, and Embase databases on May 21, 2018, using the search strings ((intervention OR programme OR program OR evaluation) AND (wash OR water OR sanitation OR hygiene)) AND ((diarrhea OR diarrhoea OR “diarrheal disease” OR “diarrhoeal disease” OR growth OR anthropometry OR anthropometrics OR HAZ OR LAZ OR “height-for-age” OR “height for age” OR “length-for-age” OR “length for age”) AND (child OR children OR infant)), further specified in the appendix (p 1). We included published studies, studies with a published protocol, and studies identified from conference abstracts, and all study designs. During the initial title or abstract screening, studies were included for further review if they measured diarrhoeal disease prevalence or child linear growth. Studies were excluded if they were set in high-income countries; were done in the public domain (ie, schools, hospitals, or child-care centres); did not include data on children under the age of 5 years; or pertained to studies that had no original data collection. For the diarrhoea analysis, all diarrhoea definitions were eligible, but because of the risk of recall bias studies were excluded if they used a recall period longer than 7 days.^23^ For the linear growth analyses, cross-sectional studies were excluded, because of the absence of longitudinal environmental contamination data measured before the growth outcomes. After reviewing the titles and abstracts, full texts were reviewed against the same inclusion and exclusion criteria. Studies were excluded during full-text review if they did not measure concentrations of faecal indicator bacteria along at least one common faecal-oral transmissions pathways (ie, drinking water, soil, or food, on children's hands or fomites, or flies in food preparation areas). Faecal indicator bacterial concentrations are less commonly measured for flies, so we included studies that measured fly density with fly traps in food preparation areas over a period of time (typically 24 h) as a proxy for the transmission of faecal contamination on to food.

Title and abstract screening and full-text reviews were duplicated by FGBG and a research assistant. The search was done in English, but full texts published in French, Portuguese, and Spanish were also reviewed. For studies determined to be eligible after full-text review, we sought IPD from the corresponding authors and extracted relevant summary information from the manuscripts. Studies for which IPD were not available were not included in parallel, conventional meta-analyses, because studies typically did not report associations between faecal exposure and health outcomes. This study was registered with PROSPERO. The study protocol with a pre-specified analysis plan was made publicly available on the Open Science Framework before beginning the data analysis, along with any updates to the search strategy and analysis plan since publication of the original protocol.

### Data analysis

We requested caregiver-reported diarrhoea and height and length measurements at the individual level, along with data for child age, sex, and survey date. At the household level we requested measurements of faecal indicator bacteria in drinking water, soil, food, fomites, and hand rinses, as well as fly densities in food preparation areas, treatment status, and date of the environmental sample collection. These variables reflect the expected common pathways of faecal exposure.^18^ As clustering variables, we requested unique identifiers for each child, household, and community to allow for adjustment of clustered health outcomes. We received anonymised data with identifying information removed and thus did not require additional institutional review board (IRB) approval for this study. Protocols for original studies were approved by local IRBs and participants provided informed consent. We extracted information on study location, study design, faecal indicator bacteria used, and whether study communities were urban or rural from study protocols or manuscripts. To control for the effects of precipitation on exposure,^24^ our model included a term differentiating between wet and dry season months based on the 30-year average monthly precipitation for each included study from the WorldClim dataset.^25^

We then matched household-level faecal contamination data to individual-level health data for all children younger than 5 years. For the diarrhoea analyses, single timepoint environmental samples collected on the same day or up to 7 days before diarrhoea data collection were matched to caregiver-reported diarrhoea. For the linear growth analyses, we matched all available environmental samples collected during the child's life up to the day that the anthropometric measurements were taken and calculated the median. We transformed faecal indicator bacteria concentrations and fly densities into categorical variables based on a log_10_ scale with four levels of contamination as our exposure variables: fewer than 1, 1–10, 11–100, and more than 100 colony-forming units (CFU) or most probable number for faecal indicator bacteria (per 100 mL for hand and fomite rinses and drinking water; per dry g for food; and per dry mg for soil); and fewer than 1, 1–10, 11–100 and more than 100 flies per 24 h for fly densities. We generated height-for-age Z-scores (HAZ) with the height, length, age, and sex data using WHO growth standards.^26^ For precipitation, we classified a month as a wet-season month if the average precipitation was more than 60 mm, and as a dry-season month if the average precipitation was less than 60 mm, based on the Köppen–Geiger climate classification system.^27^

We did our analyses using multilevel generalised mixed-effects models. Primary outcomes were diarrhoea and HAZ scores, and the parameters of interest were the odds ratio (OR) for diarrhoea and difference in HAZ scores associated with a 1-log_10_ difference in measures of faecal contamination along the different transmission pathways, indexed by p below. We also included the odds of stunting (HAZ score <2) as a secondary outcome (not pre-specified). We modelled each study individually using the following models for the primary outcomes:

$$\text{Logit}\left(d_{p,ijk}\right)=u_{ijk}+u_{jk}+u_{k}+\beta_{1}{\text{FC}}_{p,jk}+\beta_{2}{\text{Age}}_{ijk}+\beta_{3}{\text{Treat}}_{jk}+\beta_{4}{\text{Resid}}_{k}+\beta_{5}{\text{Season}}_{k}$$

$${\text{haz}}_{p,ijk}=u_{jk}+u_{k}+\beta_{1}{\text{FC}}_{p,jk}+\beta_{2}{\text{Age}}_{ijk}+\beta_{3}{\text{Treat}}_{jk}+\beta_{4}{\text{Resid}}_{k}$$

*FC*_p,jk_ represented the log_10_ categories of faecal contamination (0, 1, 2 and 3). We controlled for child age (Age_ijk_ in years), treatment status (Treat_jk_, any intervention *vs* no intervention), and residence (Resid_k_, urban *vs* rural).

For the diarrhoea model we also controlled for season (Season_k_, wet *vs* dry). In studies that had multiple children within households or communities, we controlled for clustering *u* at household-level *j* and community-level *k.* For the diarrhoea model we also controlled for clustering *u* at child-level *i* in longitudinal studies that had repeated matched individual-level diarrhoea reports and environmental samples. In the diarrhoea model, β represented the log odds of diarrhoea; in the linear growth model it represented the difference in the HAZ score. We combined effect estimates from each study in a meta regression using a random-effects model to account for between-study heterogeneity. We characterised between-study heterogeneity using *I*^2^ to describe the percentage of total variation across studies that was due to heterogeneity rather than chance.^28^

We did subgroup analyses for pathways with sufficient data by stratifying the analyses by child age, by each log_10_ category compared to no contamination, treatment status, type of faecal indicator bacteria, and urban versus rural communities. Age stratification was based on guidance from the US Environmental Protection Agency:^29^ birth to less than 3 months, 3 months to less than 6 months, 6 months to less than 12 months, 1 year to less than 2 years, and 2 years to less than 5 years. Stratification by log_10_ category and type of faecal indicator bacteria was not pre-specified in our analysis plan and was added to test how the type of faecal indicator bacteria modified the exposure–outcome associations, and to compare effect sizes for different levels of faecal contamination with no measured contamination. For the diarrhoea analyses, we also stratified by wet versus dry season and timing of environmental and diarrhoea data collection. We stratified the timing of data collection by cross-sectional (diarrhoea and environmental data collected on the same day) versus prospective (diarrhoea data collected 1–7 days after environmental data).

We did sensitivity analyses to consider the effects that our statistical analysis decisions had on the study findings. First, we repeated the analyses by both expanding the exposure variables to six log_10_ categories (<1, 1–10, 11–100, 101–1000, 1001–10 000, and >10 000) and by using a continuous log_10_ transformation. Second, to consider the effects of uncontrolled confounding, we compared findings from the original models to those that adjusted for additional potential confounders (asset index, mother's education, and household food security) for the two studies for which we were able to secure these covariates. We used the same statistical analysis methodology for all subgroup and sensitivity analyses as we did for the main outcomes, except for age and log_10_ category stratification for which we pooled data from all studies in the same model because of data sparsity in some strata in select studies. All analyses were completed in R version 3.6 using the lme4 and meta packages.^30^

We assessed the risk of bias for the participating studies for each outcome and faecal-oral transmission pathway separately. We used a modified version of the Liverpool Quality Assessment Tool, an adaptation of the Newcastle-Ottowa scale,^31^ because it was adaptable to different study designs and considers the risk of bias in both exposure and outcome measures.^32^ We assessed risk of selection, response rate, and follow-up bias by examining how households were chosen for environmental sample collection and the integrity of collected environmental data. We also assessed the risk of bias from data collection methods for exposure and outcome assessment, and from blinding.

### Role of the funding source

There was no funding source for this study. The corresponding author had full access to all the data in the study and had final responsibility for the decision to submit for publication.

## Results

**Figure 1:**
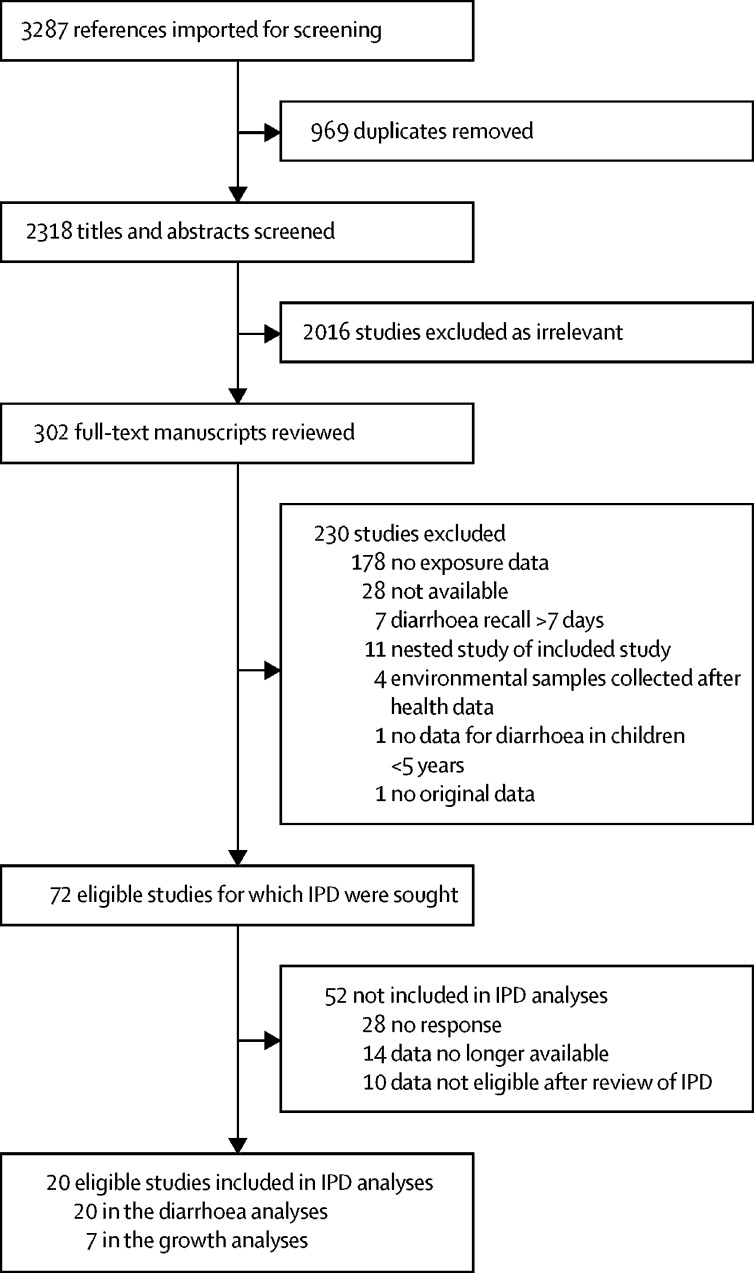
Study selection IPD=individual participant data.

We screened 2318 studies and sought IPD from 72 eligible studies (figure 1). We received IPD from 30 studies, ten of which did not meet all eligibility criteria after review of the data. 20 studies were included in the meta-analyses,^8^, ^10^, ^13^, ^33^, ^34^, ^35^, ^36^, ^37^, ^38^, ^39^, ^40^, ^41^, ^42^, ^43^, ^44^, ^45^, ^46^, ^47^, ^48^, ^49^ of which all had diarrhoea data and seven of which had linear growth data (table; appendix pp 3–7). Of the studies that we were not able to acquire IPD for, only six estimated faecal contamination along pathways other than drinking water and eight measured linear growth. Our final dataset included results for 29 548 separate individuals. We matched health-outcome observations (diarrhoea or linear growth) to faecal contamination data for drinking water (n=54 225), children's hand rinses (n=10 732), kitchen flies (n=10 514) and fomites (n=5913). Faecal contamination on fomites was characterised using sentinel toy rinses. Toys are a fomite that young children readily interact with and thus are used as a proxy for fomite faecal contamination.^15^ For food and soil, we received data from only one study, so we were not able to conduct pooled meta-analyses for these variables. We received data from countries in South America, sub-Saharan Africa, south Asia, and southeast Asia. Most of the data originated from rural settings and the faecal indicator bacteria used were *E coli* and faecal coliforms, both of which are designated by WHO as indicators for faecal contamination.^50^

**Table:** Study summary characteristics

	**Study design**	**Intervention study**	**Country**	**Rural or urban**	**Faecal indicator bacteria**	**Data availability by pathway**					
						Water	Children's hands	Fomites	Flies	Food	Soil
**Diarrhoea analyses**											
Arnold et al (2010)^33^	Cohort	Yes	India	Rural	*Escherichia Coli*	Yes	..	..	..	..	..
Benjamin-Chung et al (2018)^34^	RCT	No	Bangladesh	Rural	*E Coli*	Yes	..	Yes	Yes	..	..
Boisson et al (2010)^35^	RCT	No*	DR Congo	Rural	Faecal coliform	Yes	..	..	..	..	..
Boisson et al (2013)^36^	RCT	Yes	India	Rural and urban	Faecal coliform	Yes	..	..	..	..	..
Brown et al (2008)^37^	RCT	Yes	Cambodia	Rural	*E Coli*	Yes	..	..	..	..	..
Clasen et al (2005)^38^	RCT	Yes	Colombia	Rural	Faecal coliform	Yes	..	..	..	..	..
Clasen et al (2014)^8^	RCT	Yes	India	Rural	Faecal coliform	Yes	..	..	Yes	..	..
Davis et al (unpublished data)	RCT	Yes	Tanzania	Rural and urban	*E Coli*	Yes	..	..	..	..	..
Devamani et al (2014)^40^	Cross-sectional	Yes	Mozambique	Urban	*E Coli*	..	Yes	..	..	..	..
Ercumen et al (2015)^41^	RCT	Yes	Bangladesh	Rural	*E Coli*	Yes	..	..	..	..	..
Kirby et al (2017)^42^	Cohort	Yes	Rwanda	Rural	Faecal coliform	Yes	..	..	..	..	..
Kirby, Nagel, et al (2019)^43^	RCT	Yes	Rwanda	Rural	Faecal coliform	Yes	..	..	..	..	..
Luby et al (2015)^44^	Cohort	Yes	Bangladesh	Rural	*E Coli*	Yes	..	..	..	..	..
Patil et al (2014)^10^	RCT	Yes	India	Rural	*E Coli*	Yes	..	..	..	..	..
Peletz et al (2011)^45^	Cross-sectional	No	Zambia	Rural	Faecal coliform	Yes	..	..	..	..	..
Peletz et al (2012)^46^	RCT	Yes	Zambia	Peri-urban†	Faecal coliform	Yes	..	..	..	..	..
Pickering, Ercumen, et al (2018)^47^	RCT	Yes	Bangladesh	Rural	*E Coli*	Yes	Yes	..	Yes	Yes	Yes
Pickering et al (2019)^48^	RCT	Yes	Kenya	Rural	*E Coli*	Yes	Yes	Yes	Yes	..	..
Reese et al (2019)^13^	Cohort	Yes	India	Rural	*E Coli*	Yes	Yes	..	..	..	..
Sinharoy et al (2017)^49^	RCT	Yes	Rwanda	Rural	Faecal coliform	Yes	..	..	..	..	..
**Growth analyses**											
Arnold et al (2010)^33^	Cohort	Yes	India	Rural	*E Coli*	Yes	..	..	..	..	..
Clasen et al (2014)^8^	RCT	Yes	India	Rural	Faecal coliform	Yes	..	..	Yes	..	..
Patil et al (2014)^10^	RCT	Yes	India	Rural	*E Coli*	Yes	..	..	..	..	..
Pickering, Ercumen, et al (2018)^47^	RCT	Yes	Bangladesh	Rural	*E Coli*	Yes	Yes	Yes	Yes	Yes	Yes
Pickering et al (2019)^48^	RCT	Yes	Kenya	Rural	*E Coli*	Yes	Yes	Yes	Yes	..	..
Reese et al (2019)^13^	Cohort	Yes	India	Rural	*E Coli*	Yes	Yes	..	..	..	..
Sinharoy et al (2017)^49^	RCT	Yes	Rwanda	Rural	Faecal coliform	Yes	..	..	..	..	..

RCT=randomised controlled trial.

* Only data at baseline used (no intervention implemented)—no usable exposure data during trial.

† Classified as urban in our analysis.

**Figure 2:**
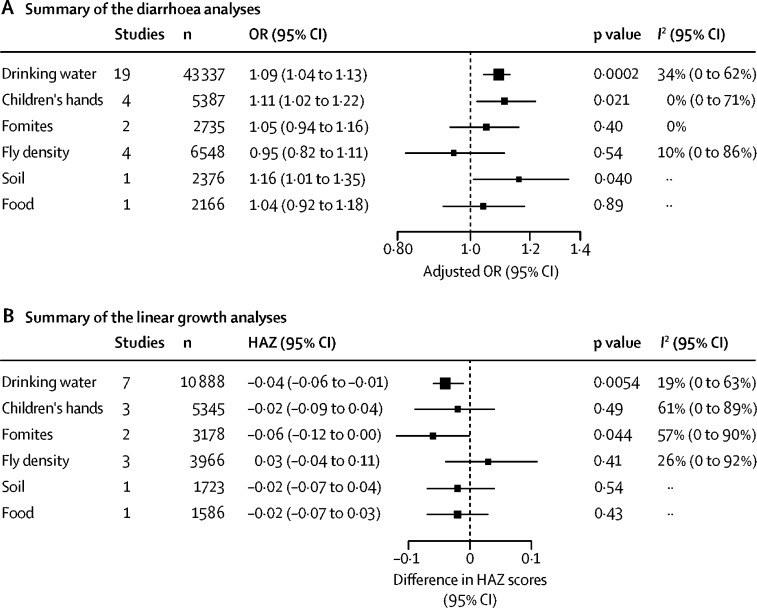
Summary of analyses for diarrhoea (A) and difference in height-for-age Z scores (linear growth; B). Data are for 1-log higher faecal indicator bacteria concentrations in drinking water, on children's hands and on fomites, in food and in soil, and for 1-log higher concentration in kitchen fly density. OR=odds ratio. HAZ=height-for-age Z-scores.

**Figure 3:**
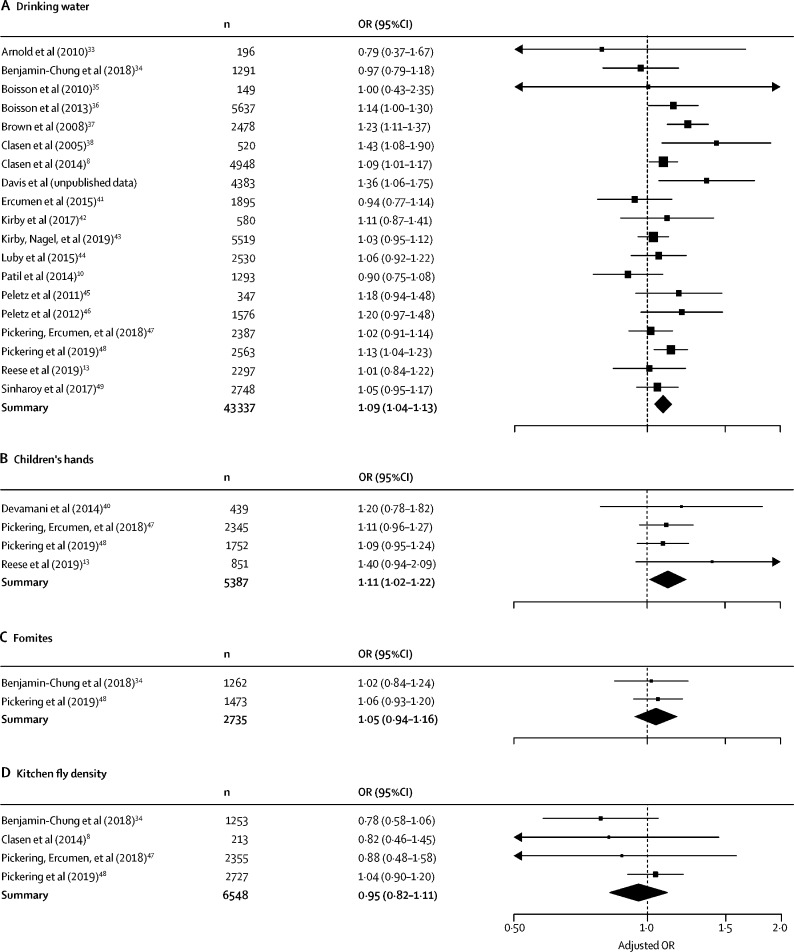
Forest plot of diarrhoea analyses Data are for 1-log higher faecal indicator bacteria concentrations in (A) drinking water, (B) on children's hands, (C) on fomites, and (D) for 1-log higher concentration in kitchen fly density. OR=odds ratio.

**Figure 4:**
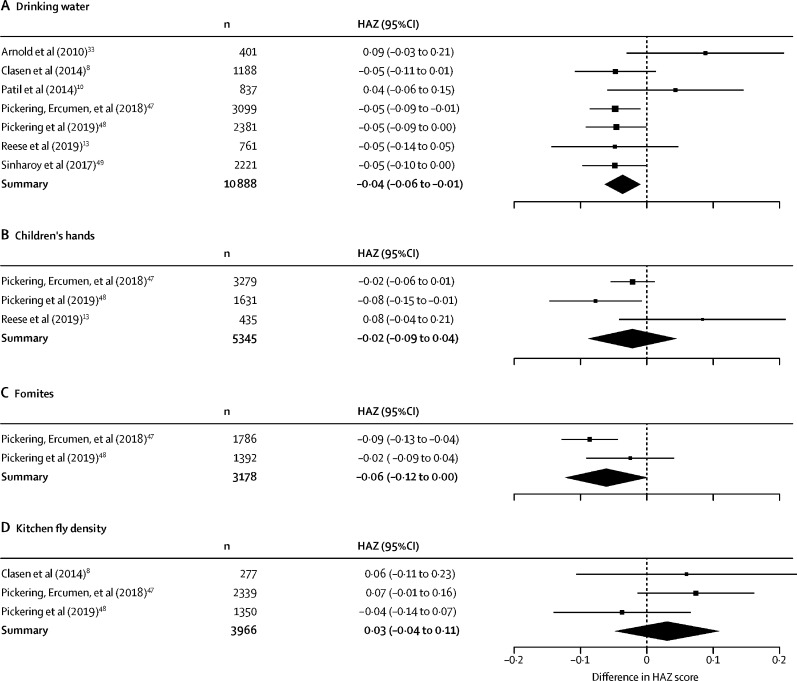
Forest plot of linear growth analyses Data are differences in HAZ scores for 1-log higher median faecal indicator bacteria concentrations in (A) drinking water, (B) on children's hands, (C) and on fomites, and (D) for 1-log higher concentration in median kitchen fly density. HAZ=height-for-age *Z* scores.

We found higher odds of diarrhoea with 1-log_10_ higher faecal indicator bacteria concentrations in drinking water and on child hands. There was no evidence that faecal indicator bacteria on fomites or food preparation area fly density was associated with diarrhoea (Figure 2, Figure 3). For the linear growth analyses, matched median faecal contamination was derived from 1–8 samples, depending on data availability. We found lower HAZ scores with 1-log_10_ higher median faecal indicator bacteria concentrations in drinking water and on fomites. There was no evidence that faecal indicator bacteria on child hands or food preparation area fly density was associated with linear growth (Figure 2, Figure 4).

For child stunting, we found higher odds of stunting with 1-log_10_ higher median faecal indicator bacteria concentrations on children's hands (OR 1·08; 95% CI 1·02–1·15; p=0·0055; *I*^2^=0%) and fomites (OR 1·10; 95% CI 1·03–1·19; p=0·0086; *I*^2^=0%). There was weaker evidence for an association between median faecal indicator bacteria concentrations in drinking water and stunting (OR 1·05, 95% CI 1·00–1·11; p=0·062, *I*^2^=30%, 95% CI 0–70; appendix p 10).

Secondary analyses suggested that the higher odds of diarrhoea with faecal indicator bacteria concentrations in drinking water and on children's hands, and the lower HAZ scores with faecal indicator bacteria concentrations in drinking water and on fomites was driven by concentrations of measured faecal indicator bacteria above 10 CFU or most probable number per 100 mL sample and was less evident for 1–10 CFU or most probable number (appendix pp 11–12). Age-stratified analyses of the pooled data suggested that the higher odds of diarrhoea associated with higher faecal indicator bacteria concentrations in drinking water might be less evident in children under the age of 6 months (appendix pp 8–9). For children's hands and fomite rinses, higher odds of diarrhoea were observed for children age 12–24 months. For child linear growth, we found lower HAZ scores for children age 6–24 months and age above 12 months with higher median faecal indicator bacteria concentrations in drinking water and on fomites, respectively. Other stratified analyses suggested that there might be a stronger relationship between faecal contamination in water and diarrhoea in urban communities compared with rural communities (OR 1·25, 95% CI 1·12–1·40 *vs* 1·07, 1·02–1·11). We found no other differences by treatment status, type of faecal indicator bacteria used, urban versus rural communities, dry versus wet season, and prospective versus cross-sectional diarrhoea data collection.

Sensitivity analyses suggested similar findings after using different transformations of exposure variables using the six log_10_ categories and continuous log_10_ (appendix pp 13–14). Confounding sensitivity analyses using data from two included studies indicated similar findings for the diarrhoea analyses comparing the original models to those that adjusted for additional covariates, whereas there was some difference in findings for the linear growth analyses (appendix pp 14–15).

Findings from our risk of bias assessment suggested that high risk of bias for both the diarrhoea and linear growth analyses originated from the outcome assessments, mainly because it is not possible to mask respondents or data collection staff from most WaSH interventions (appendix pp 16–17). This might introduce bias to the analyses, for example if interventions reduced levels of household faecal contamination and members of those households were also less likely to report diarrhoea. Our analyses also included studies that carried possible risks of selection, response rate and follow-up bias, primarily where environmental assessments were tertiary outcomes, so study design was not tailored towards optimal environmental sample collection.

## Discussion

By contrast with mixed results from individual studies and meta-analyses using summary estimates, our analysis of nearly 90 000 IPD points from 20 studies suggests that household faecal indicator bacteria concentrations are associated with child diarrhoea and impaired linear growth. Evidence of associations varied by exposure pathway as did data availability, with faecal indicator bacteria concentrations in drinking water representing more than half the included environmental data, whereas food and soil were sampled in only one study. We were not able to control for all potential confounders, so our findings should be interpreted with caution. We also found low to moderate between-study heterogeneity in some meta-analyses. These results are the first to pool data from a number of settings to show that faecal contamination in the household environment is associated with impaired child linear growth, and that faecal contamination along pathways other than drinking water can have adverse effects on child health. The findings support a recent consensus statement by WaSH researchers recommending that interventions should focus more closely on the need to reduce faecal contamination in the domestic environment to achieve consistent child health benefits.^51^

In our age-stratified analyses, we found no association between faecal indicator bacteria concentrations in drinking water and diarrhoea for children under the age of 6 months, possibly because infants have little exposure to household drinking water before weaning.^52^ As a proxy for exposure from contaminated hand-mouthing and object-mouthing, we found the strongest associations between fomite rinses and diarrhoea and linear growth, and hand rinses and diarrhoea, among children age 12–24 months. In other stratified analyses we found no differences by faecal indicator bacteria used, by contrast with a previous systematic review that found a significant association between levels of *E coli* in drinking water and diarrhoea but not for faecal coliforms.^20^ We found that overall associations were driven by high levels of faecal contamination, but this finding could partly be induced by limits of detection at low levels of faecal contamination, leading to misclassifications of exposure.

The limitations of our study reflect shortcomings in exposure assessment in WaSH research. First, despite the large number of studies attempting to evaluate the effect of WaSH on health, few actually measure faecal contamination in the environment, despite the fact that faecal exposure is a necessary intermediate step along the pathway to adverse health effects. The studies that do are largely confined to measurements of faecal contamination in drinking water despite evidence suggesting that food and exposures along other pathways, such as soil, are likely contributors to overall faecal exposure.^53^, ^54^ Second, we were not able to obtain sufficient IPD for some of the pre-specified stratified analyses. We found a stronger effect from faecal contamination in drinking water on child diarrhoea in urban areas, but most available datasets had limited or no IPD for urban settings, except for drinking water and diarrhoea and a small amount of urban data for child hands and diarrhoea. Understanding the relationship between faecal contamination and child health is a priority in rapidly urbanising low-income countries. We also had few data for young children under the age of 6 months, resulting either in no estimates or estimates with high levels of uncertainty for this age group. Third, there is risk of bias from the outcome assessments because of reliance on caregiver-reported diarrhoea and anthropometric measurements. Reported diarrhoea is subject to courtesy and recall bias,^23^, ^55^ and anthropometric measurements are also prone to measurement error, particularly when data collection staff are not masked to interventions.^56^ Fourth, studies generally use faecal indicator bacteria concentrations as a proxy for health-related faecal contamination, an indicator with well documented shortcomings.^57^ Fifth, none of the included studies estimated faecal contamination along all included pathways, so we were limited to modelling each pathway individually without adjusting for faecal contamination along the other pathways in our models. Although these factors limit the inferences that can be drawn from the data, they also suggest the need for significant improvements in the manner in which WaSH research assesses the effect of interventions on faecal exposure.

Even though our findings provide evidence of associations between faecal contamination and adverse child health outcomes, we were not able to control for all possible factors such as socioeconomic status and use of medication that could affect the modelled exposure-outcome associations, so there is risk of uncontrolled confounding in our results. Additionally, we used measured household-level faecal indicator bacteria concentrations and fly density as proxies for exposure with no data on the extent to which individuals were actually exposed to specific disease-causing pathogens. These types of proxy measures are prone to introducing heterogeneity to exposure-outcome effect estimates as well as bias to the associations, commonly towards the null.^58^ So although we found associations between domestic faecal contamination and adverse child health outcomes, improved measures of exposure—including direct measures of known or suspected enteric pathogens—would better characterise exposure-outcome relationships, and could inform intervention design and evaluation through this characterisation.
