# Supplementary material for: Faecal contamination of the environment and child health: a systematic review and individual participant data meta-analysis
Source: Lancet Planet Health. 2020 Sep 9;4(9):e405–15. doi: 10.1016/S2542-5196(20)30195-9 (PMC7653404; doi:10.1016/S2542-5196(20)30195-9)
Supplement: Supplementary appendix [file mmc1.pdf]

THE LANCET
Planetary Health

Supplementary appendix

This appendix formed part of the original submission and has been peer reviewed. We post it as supplied by the authors.

Supplement to: Goddard FGB, Pickering AJ, Ercumen E, Brown J, Chang HH, Clasen T. Faecal contamination of the environment and child health: a systematic review and individual participant data meta-analysis. *Lancet Planet Health* 2020; **4:** e405–15.

Appendix A - Search Strings

Generic Search String: ((intervention OR programme OR program OR evaluation) AND (wash OR water OR sanitation OR hygiene)) AND ((diarrhea OR diarrhoea OR “diarrheal disease” OR “diarrhoeal disease” OR growth OR anthropometry OR anthropometrics OR HAZ OR LAZ OR “height-for-age” OR “height for age” OR “length-for-age” OR “length for age”) AND (child OR children OR infant))

PubMed: ((intervention[tw] OR programme[tw] OR program[tw] evaluation[tw]) AND (wash[tw] OR water[tw] OR sanitation[tw] OR hygiene[tw])) AND ((diarrhea[tw] OR diarrhoea[tw] OR “diarrheal disease”[tw] OR “diarrhoeal disease”[tw] OR growth[tw] OR anthropometry[tw] OR anthropometrics[tw] OR HAZ[tw] OR LAZ[tw] OR “height-for-age”[tw] OR “height for age”[tw] OR “length-for-age”[tw] OR “length for age”[tw]) AND (child[tw] OR children[tw] OR infant[tw]))

Web of Science: TS = ((intervention OR programme OR program OR evaluation) AND (wash OR water OR sanitation OR hygiene)) AND TS = ((diarrhea OR diarrhoea OR "diarrheal disease" OR "diarrhoeal disease" OR growth OR anthropometry OR anthropometrics OR HAZ OR LAZ OR "height-for-age" OR "height for age" OR "length-for-age" OR "length for age") AND (child OR children OR infant))

EMBASE: ((intervention:ti,ab,kw OR programme:ti,ab,kw OR program:ti,ab,kw OR evaluation:ti,ab,kw) AND (wash:ti,ab,kw OR water:ti,ab,kw OR sanitation:ti,ab,kw OR hygiene:ti,ab,kw)) AND ((diarrhea:ti,ab,kw OR diarrhoea:ti,ab,kw OR ‘diarrheal disease’:ti,ab,kw OR ‘diarrhoeal disease’:ti,ab,kw OR growth:ti,ab,kw OR anthropometry:ti,ab,kw OR anthropometrics:ti,ab,kw OR HAZ:ti,ab,kw OR LAZ:ti,ab,kw OR ‘height-for-age’:ti,ab,kw OR ‘height for age’:ti,ab,kw OR ‘length-for-age’:ti,ab,kw OR ‘length for age’:ti,ab,kw) AND (child:ti,ab,kw OR children:ti,ab,kw OR infant:ti,ab,kw))

Appendix B – Supplementary Tables and Figures

Tables

Table S1: Median fecal indicator bacteria concentrations and diarrhea prevalence, stratified by treatment status, for studies included in the drinking water-diarrhea meta-analyses

Study ID	Treatment status	Intervention type	N	Median drinking water fecal indicator bacteria concentration, CFU/MPN[1] per 100ml (IQR)	Diarrhea prevalence (%)	Diarrhea definition	Recall period (days)
Arnold, 2010	Intervention	Water, Sanitation, Hygiene	100	70 (325)	2.0	WHO-defined[2] or 1 or more stools with blood in 24 hours	7
	Control		126	100 (300)	1.6		
Benjamin-Chung, 2018	No intervention		1,349	5 (43)	8.3	WHO-defined or 1 or more stools with blood in 24 hours	7
Boisson, 2010	No intervention		184	720 (2,850)	22.3	WHO-defined	7
Boisson, 2013	Intervention	Water	3,345	68 (1,060)	2.9	WHO-defined	3
	Control		3,283	196 (1,892)	3.0		
Brown, 2008	Intervention	Water	1,872	5 (50)	17.0	WHO-defined or 1 or more stools with blood in 24 hours	7
	Control		923	650 (1400)	24.1		
Clasen, 2005	Intervention	Water	380	2 (25)	15.8	WHO-defined	7
	Control		231	102 (276)	26.8		
Clasen, 2014	Intervention	Sanitation	2,880	60 (660)	8.8	WHO-defined	7
	Control		2,857	60 (560)	9.8		
Davis, in prep.	Intervention	Water, Hygiene	2,675	20 (59)	1.6	WHO-defined	2
	Control		1,946	42 (160)	4.1		
Ercumen, 2015	Intervention	Water	1,278	0 (5)	6.9	WHO-defined	7
	Control		646	16 (70)	10.4		
Kirby, 2017	Intervention	Water	240	0 (1)	13.3	WHO-defined	7
	Control		340	4 (28)	19.7		
Kirby, Nagel, 2019	Intervention	Water, Air	2,649	2 (52)	10.5	WHO-defined	7
	Control		2,870	14 (178)	12.8		
Luby, 2015	Intervention	Water, Sanitation, Hygiene	1,405	2 (24)	10.0	WHO-defined	2
	Control		1,125	2 (22)	9.2		
Patil, 2015	Intervention	Sanitation	641	34 (199)	7.8	WHO-defined or 1 or more stools with blood in 24 hours	7
	Control		652	60 (196)	9.5		
Peletz, 2011	No intervention		387	64 (500)	20.9	WHO-defined	7
Peletz, 2012	Intervention	Water	961	0 (4)	4.4	WHO-defined	7
	Control		887	230 (1,232)	8.9		
Pickering, Ercumen, 2018	Intervention	Water, Sanitation, Hygiene	1,597	1 (10)	17.7	WHO-defined	7
	Control		790	4 (41)	17.8		
Pickering, 2019	Intervention	Water, Sanitation, Hygiene	1,644	16 (99)	29.9	WHO-defined	7
	Control		919	32 (134)	26.2		
Reese, 2019	Intervention	Water, Sanitation	1,072	10 (49)	5.0	WHO-defined	7
	Control		1,268	15 (56)	5.0		
Sinharoy, 2017	Intervention	Water, Sanitation, Hygiene	2,017	14 (162)	9.9	WHO-defined	7
	Control		1,042	12 (114)	11.4		

[1]CFU-Colony Forming Unit; MPN-Most Probable Number

[2]Three or more loose or watery stools in a 24 hour period

Table S2: Median fecal indicator bacteria concentrations and diarrhea prevalence, stratified by treatment status, for studies included in the child hands-diarrhea meta-analyses

Study ID	Treatment status	Intervention type	N	Median child hand rinse fecal indicator bacteria concentration, CFU/MPN[1] per 100ml (IQR)	Diarrhea prevalence (%)	Diarrhea definition	Recall period (days)
Devamani, 2014	Intervention	Water, Sanitation	239	6 (21)	7.5	WHO-defined[2] or 1 or more stools with blood in 24 hours	7
	Control		200	4 (21)	28.7		
Pickering, Ercumen, 2018	Intervention	Water, Sanitation, Hygiene	1,566	1 (3)	17.9	WHO-defined	7
	Control		779	1 (5)	17.8		
Pickering, 2019	Intervention	Water, Sanitation, Hygiene	847	88 (488)	30.5	WHO-defined	7
	Control		905	66 (526)	26.4		
Reese, 2019	Intervention	Water, Sanitation	400	16 (40)	4.2	WHO-defined	7
	Control		451	19 (51)	4.9		

[1]CFU-Colony Forming Unit; MPN-Most Probable Number
[2]Three or more loose or watery stools in a 24 hour period

Table S3: Median fecal indicator bacteria concentrations and diarrhea prevalence, stratified by treatment status, for studies included in the fomites-diarrhea meta-analyses

Study ID	Treatment status	Intervention type	N	Median fomite rinse fecal indicator bacteria concentration, CFU/MPN[1] per 100ml (IQR)	Diarrhea prevalence (%)	Diarrhea definition	Recall period (days)
Benjamin-Chung, 2018	No intervention		1,262	30 (189)	8.4	WHO-defined[2] or 1 or more stools with blood in 24 hours	7
Pickering, 2019	Intervention	Water, Sanitation, Hygiene	687	3 (13)	29.7	WHO-defined	7
	Control		786	2 (13)	26.0		

[1]CFU-Colony Forming Unit; MPN-Most Probable Number
[2]Three or more loose or watery stools in a 24 hour period

Table S4: Median kitchen fly density and diarrhea prevalence, stratified by treatment status, for studies included in the flies-diarrhea meta-analyses

Study ID	Treatment status	Intervention type	N	Median kitchen fly density per 24 hours (IQR)	Diarrhea prevalence (%)	Diarrhea definition	Recall period (days)
Benjamin-Chung, 2018	No intervention		1,253	3 (10)	8.1	WHO-defined[1] or 1 or more stools with blood in 24 hours	7
Clasen, 2014	Intervention	Sanitation	98	9 (34)	10.2	WHO-defined	7
	Control		115	5 (25)	7.0		
Pickering, Ercumen, 2018	Intervention	Water, Sanitation, Hygiene	1,567	0 (0.2)	17.8	WHO-defined	7
	Control		790	0 (0.1)	17.5		
Pickering, 2019	Intervention	Water, Sanitation, Hygiene	1,502	2 (5)	29.2	WHO-defined	7
	Control		1,225	1 (4)	26.0		

[1]Three or more loose or watery stools in a 24 hour period

Table S5: Median fecal indicator bacteria concentrations and diarrhea prevalence, stratified by treatment status, for studies included in the soil-diarrhea analysis

Study ID	Treatment status	Intervention type	N	Median soil fecal indicator bacteria concentration, CFU/MPN[1] per dry mg (IQR)	Diarrhea prevalence (%)	Diarrhea definition	Recall period (days)
Pickering, Ercumen, 2018	Intervention	Water, Sanitation, Hygiene	1,586	148 (856)	17.7	WHO-defined[2]	7
	Control		787	193 (1,520)	17.5		

[1]CFU-Colony Forming Unit; MPN-Most Probable Number
[2]Three or more loose or watery stools in a 24 hour period

Table S6: Median fecal indicator bacteria concentrations and diarrhea prevalence, stratified by treatment status, for studies included in the food-diarrhea analysis

Study ID	Treatment status	Intervention type	N	Median food fecal indicator bacteria concentration, CFU/MPN[1] per dry g (IQR)	Diarrhea prevalence (%)	Diarrhea definition	Recall period (days)
Pickering, Ercumen, 2018	Intervention	Water, Sanitation, Hygiene	1,449	1 (19)	17.9	WHO-defined[2]	7
	Control		718	1 (14)	18.1		

[1]CFU-Colony Forming Unit; MPN-Most Probable Number
[2]Three or more loose or watery stools in a 24 hour period

Table S7: Median fecal indicator bacteria concentrations and HAZ scores, stratified by treatment status, for studies included in the drinking water-linear growth meta-analyses

Study ID	Treatment status	Intervention type	N	Median drinking water fecal indicator bacteria concentrations, CFU/MPN[1] per 100ml (IQR)	Median time period between samples, in months (IQR)[2]	Median HAZ score (IQR)
Arnold, 2010	Intervention	Water, Sanitation, Hygiene	189	100 (300)	4.8 (3.0)	-1.73 (1.54)
	Control		212	100 (323)	4.7 (3.2)	-1.90 (1.64)
Clasen, 2014	Intervention	Sanitation	612	194 (1,105)	6.4 (4.8)	-1.49 (1.65)
	Control		576	240 (1,300)	6.3 (5.2)	-1.35 (1.73)
Patil, 2015	Intervention	Sanitation	400	35 (199)	Not applicable	-1.91 (1.87)
	Control		437	61 (197)	Not applicable	-2.20 (1.90)
Pickering, Ercumen, 2018	Intervention	Water, Sanitation, Hygiene	1,851	1 (16)	8.9 (2.6)	-1.74 (1.37)
	Control		1,248	6 (50)	8.7 (2.2)	-1.64 (1.35)
Pickering, 2019	Intervention	Water, Sanitation, Hygiene	1,545	27 (119)	12.2 (1.1)	-1.45 (1.40)
	Control		836	51 (166)	12.0 (1.1)	-1.47 (1.46)
Reese, 2019	Intervention	Water, Sanitation	341	20 (48)	4.5 (0.9)	-1.60 (1.38)
	Control		420	25 (60)	4.4 (0.9)	-1.79 (1.57)
Sinharoy, 2017	Intervention	Water, Sanitation, Hygiene	1,491	18 (212)	28.6 (1.6)	-1.74 (1.49)
	Control		772	12 (112)	28.4 (1.0)	-1.75 (1.55)

1 CFU-Colony Forming Unit; MPN-Most Probable Number
[2]For children with more than one matched sample

Table S8: Median fecal indicator bacteria concentrations and HAZ scores, stratified by treatment status, for studies included in the child hands-linear growth meta-analyses

Study ID	Treatment status	Intervention type	N	Median child hand fecal indicator bacteria concentrations, CFU/MPN[1] per 100ml (IQR)	Median time period between samples, in months (IQR) [2]	Median HAZ score (IQR)
Pickering, Ercumen, 2018	Intervention	Water, Sanitation, Hygiene	2,031	60 (749)	8.9 (2.6)	-1.76 (1.34)
	Control		1,248	101 (838)	8.7 (2.2)	-1.64 (1.35)
Pickering, 2019	Intervention	Water, Sanitation, Hygiene	790	126 (487)	12.2 (1.3)	-1.38 (1.34)
	Control		841	93 (489)	12 (1.1)	-1.48 (1.45)
Reese, 2019	Intervention	Water, Sanitation	204	20 (46)	8.8 (0.5)	-1.74 (1.41)
	Control		231	23 (60)	8.8 (0.6)	-1.85 (1.53)

[1] CFU-Colony Forming Unit; MPN-Most Probable Number
[2] For children with more than one matched sample

Table S9: Median fecal indicator bacteria concentrations and HAZ scores, stratified by treatment status, for studies included in the fomites-linear growth meta-analyses

Study ID	Treatment status	Intervention type	N	Median fomite fecal indicator bacteria concentrations, CFU/MPN[1] per 100ml (IQR)	Median time period between samples, in months (IQR) [2]	Median HAZ score (IQR)
Pickering, Ercumen, 2018	Intervention	Water, Sanitation, Hygiene	669	41 (281)	Not applicable	-1.39 (1.39)
	Control		723	25 (223)	16.3 (2.6)	-1.48 (1.43)
Pickering, 2019	Intervention	Water, Sanitation, Hygiene	935	4 (22)	12.3 (0.7)	-1.70 (1.29)
	Control		851	4 (21)	12.2 (0.7)	-1.61 (1.33)

[1] CFU-Colony Forming Unit; MPN-Most Probable Number
[2] For children with more than one matched sample

Table S10: Median kitchen fly density and HAZ scores, stratified by treatment status, for studies included in the flies-linear growth meta-analyses

Study ID	Treatment status	Intervention type	N	Median kitchen fly density per 24 hours (IQR)	Median time period between samples, in months (IQR) [1]	Median HAZ score (IQR)
Clasen, 2014	Intervention	Sanitation	141	14 (28)	0.03 (0)	-1.39 (1.71)
	Control		136	23 (65)	0.03 (0)	-1.47 (1.67)
Pickering, Ercumen, 2018	Intervention	Water, Sanitation, Hygiene	1,667	0 (1)	8.9 (2.6)	-1.70 (1.34)
	Control		1,248	0 (2)	8.8 (2.1)	-1.65 (1.35)
Pickering, 2019	Intervention	Water, Sanitation, Hygiene	1,432	2 (4)	12.2 (1.2)	-1.43 (1.39)
	Control		1,249	2(4)	12.0 (1.1)	-1.44 (1.41)

[1] For children with more than one matched sample

Table S11: Median fecal indicator bacteria concentrations and HAZ scores, stratified by treatment status, for studies included in the soil-linear growth analysis

Study ID	Treatment status	Intervention type	N	Median soil fecal indicator bacteria concentrations, CFU/MPN[1] per dry mg (IQR)	Median time period between samples, in months (IQR)	Median HAZ score (IQR)
Pickering, Ercumen, 2018	Intervention	Water, Sanitation, Hygiene	1,166	151 (838)	Not applicable	-1.75 (1.42)
	Control		557	181 (1,329)	Not applicable	-1.67 (1.39)

[1]CFU-Colony Forming Unit; MPN-Most Probable Number

Table S12: Median fecal indicator bacteria concentrations and HAZ scores, stratified by treatment status, for studies included in the food-linear growth analysis

Study ID	Treatment status	Intervention type	N	Median food fecal indicator bacteria concentrations, CFU/MPN[1] per dry g (IQR)	Median time period between samples, in months (IQR)	Median HAZ score (IQR)
Pickering, Ercumen, 2018	Intervention	Water, Sanitation, Hygiene	1,065	1 (21)	Not applicable	-1.73 (1.39)
	Control		521	1 (14)	Not applicable	-1.62 (1.35)

[1]CFU-Colony Forming Unit; MPN-Most Probable Number

Table S13: Results from stratified analyses - Diarrhea

Stratification	Drinking water	Child hands	Fomites	Flies	Food	Soil
	Odds of diarrhea and 95% CI					
**Log10 category (CFU/MPN[1] or fly density)**						
0	Reference	Reference	Reference	Reference	Reference	Reference
1-10	1·00 (0·90 – 1·11)	1·05 (0·81 – 1·35)	0·88 (0·68 – 1·14)	0·95 (0·81 – 1·12)	1·12 (0·84 – 1·53)	1·10 (0·59 – 2..06)
11-100	1·08 (0·98 – 1·19)	1·39 (1·03 – 1·89)	0·95 (0·69 – 1·30)	0·91 (0·68 – 1·21)	1·08 (0·75 – 1·55)	1·12 (0·67 – 1·87)
+100	1·30 (1·19 – 1·43)	1·43 (0·99 – 2.09)	1·12 (0·79 – 1·59)	0·57 (0·13 – 2.54)	1·17 (0·80 – 1·70)	1·43 (0·91 – 2.25)
**Age (months)**						
0-3	0·99 (0·84 – 1·16)	1·22 (0·97 – 1·53)	No data available	0·70 (0·23 – 2.11)	1·03 (0·82 – 1·28)	0·99 (0·78 – 1·24)
3-6	0·95 (0·84 – 1·07)	1·05 (0·86 – 1·27)	No data available	0·83 (0·43 – 1·62)	0·99 (0·84 – 1·18)	1·24 (1·02 – 1·51)
6-12	1·12 (1·03 – 1·21)	1·05 (0·88 – 1·27)	0·96 (0·75 – 1·23)	0·92 (0·67 – 1·26)	1·08 (0·26 – 4.40)	1·32 (0·17 – 9.96)
12-24	1·09 (1·04 – 1·15)	1·38 (1·15 – 1·65)	1·18 (1·00 – 1·23)	0·96 (0·80 – 1·15)	0·38 (0·07 – 2.13)	0·65 (0·31 – 1·37)
24-60	1·10 (1·06 – 1·15)	0·94 (0·79 – 1·13)	0·93 (0·79 – 1·10)	0·98 (0·81 – 1·19)	1·42 (0·83 – 2.43)	2.07 (0·81 – 5.29)
**Treatment Status**						
Intervention	1·11 (1·05 – 1·18)	1·10 (0·95 – 1·27)	0·92 (0·77 – 1·11)	0·93 (0·74 – 1·13)	1·06 (0·93 – 1·22)	1·18 (1·01 – 1·38)
Control	1·05 (0·99 – 1·11)	1·14 (0·99 – 1·31)	1·11 (0·95 – 1·31)	0·98 (0·75 – 1·28)	1·00 (0·83 – 1·20)	1·09 (0·89 – 1·33)
**Fecal Indicator Bacteria**						
E. Coli	1·06 (0·99 – 1·15)	All E. Coli	All E. Coli	Not applicable	All E. Coli	All E. Coli
Fecal coliform	1·09 (1·04 – 1·13)					
**Residence**						
Urban	1·25 (1·12 – 1·40)	1·20 (0·78 – 1·82)	All rural	All rural	All rural	All rural
Rural	1·07 (1·02 – 1·11)	1·11 (1·01 – 1·22)				
**Season**						
Dry	1·06 (0·99 – 1·13)	1·14 (0·98 – 1·34)	1·03 (0·78 – 1·34)	0·84 (0·35 – 2.05)	1·04 (0·89 – 1·21)	1·11 (0·96 – 1·29)
Wet	1·10 (1·04 – 1·17)	1·10 (0·99 – 1·22)	1·05 (0·94 – 1·18)	1·00 (0·84 – 1·20)	1·03 (0·87 – 1·22)	1·29 (0·98 – 1·70)
**Sample Collection**						
Prospective	1·10 (1·01 – 1·19)	1·11 (1·00 – 1·22)	1·06 (0·93 – 1·20)	1·03 (0·89 – 1·18)	1·00 (0·85 - 1·15)	1·28 (1·07 – 1·53)
Cross-sectional	1·08 (1·02 – 1·14)	1·13 (0·92 – 1·39)	1·02 (0·84 – 1·24)	0·79 (0·60 – 1·06)	1·09 (0·93 – 1·27)	1·00 (0·82 – 1·20)

[1]CFU-Colony Forming Unit; MPN-Most Probable Number

Table S14: Results from stratified analyses – Linear growth

	Difference in height-for-age Z scores and 95% CI					
**Stratification**	**Drinking water**	**Child hands**	**Fomites**	**Flies**	**Food**	**Soil**
**Log category (CFU/MPN[1] or fly density)**						
0	Reference	Reference	Reference	Reference	Reference	Reference
1-10	-0·05 (-0·11 – 0·02)	0·01 (-0·11 – 0·13)	-0·06 (-0·17 – 0·05)	0·06 (-0·02 – 0·14)	-0·20 (-0·34 – -0·07)	0·33 (0·09 – 0·57)
11-100	-0·06 (-0·12 – 0·01)	-0·02 (-0·12 – 0·09)	-0·19 (-0·30 – -0·08)	0·05 (-0·01 – 0·19)	-0·03 (-0·17 – 0·12)	0·11 (-0·10 – 0·31)
+100	-0·09 (-0·16 – -0·02)	-0·03 (-0·13 – 0·07)	-0·23 (-0·34 – -0·12)	0·24 (-0·16 – 0·64)	-0·07 (-0·22 – 0·09)	0·07 (-0·13 – 0·26)
**Age (months)**						
0-3	0·00 (-0·15 – 0·15)	No data available	No data available	No data available	No data available	No data available
3-6	-0·02 (-0·14 – 0·10)	-0·20 (-0·44 – 0·04)	No data available	-0·27 (-0·95 – 0·41)	No data available	No data available
6-12	-0·05 (-0·09 – -0·01)	-0·03 (-0·08 – 0·02)	No data available	-0·16 (-0·39 – 0·06)	-0·14 (-0·42 – 0·14)	-0·13 (-0·44 – 0·19)
12-24	-0·04 (-0·07 – -0·01)	-0·02 (-0·05 – 0·01)	-0·07 (-0·11 – -0·03)	0·12 (0·05 – 0·19)	-0·03 (-0·09 – 0·02)	-0·04 (-0·10 – 0·02)
24-60	-0·02 (-0·06 – 0·01)	-0·02 (-0·08 – 0·04)	-0·07 (-0·13 – -0·01)	0·07 (-0·02 – 0·15)	0·03 (-0·07 – 0·13)	0·08 (-0·05 – 0·22)
**Treatment Status**						
Intervention	-0·02 (-0·07 – 0·02)	-0·02 (-0·07 – 0·02)	-0·07 (-0·12 – -0·02)	0·00 (-0·08 – 0·07)	-0·02 (-0·08 – 0·04)	0·02 (-0·04 – 0·09)
Control	-0·05 (-0·09 – -0·01)	-0·03 (-0·14 – 0·08)	-0·05 (-0·15 – 0·05)	0·04 (-0·07 – 0·14)	-0·02 (-0·10 – 0·07)	-0·10 (-0·19 – -0·01)
**Fecal Indicator Bacteria**						
E. Coli	-0·02 (-0·07 – 0·02)	All E. Coli	All E. Coli	Not applicable	All E. Coli	All E. Coli
Fecal colofirm	-0·05 (-0·09 – -0·01)					
**Residence**						
Urban	All rural	All rural	All rural	All rural	All rural	All rural
Rural						

[1]CFU-Colony Forming Unit; MPN-Most Probable Number

Figures

Pathway	Studies	N	OR (95% CI)	I2 (95% CI)
Drinking water	7	10,888	1.05 (1.00, 1.11)	30% (0–70%)
Child hands	3	5,345	1.08 (1.03, 1.19)	0% (0–81%)
Fomites	2	3,178	1.10 (1.03, 1.19)	0% (–)
Fly density	3	3,966	0.95 (0.84, 1.08)	0% (0–84%)
Soil	1	1.723	0.97 (0.88, 1.08)	–
Food	1	1,586	1.07 (0.98, 1.18)	–

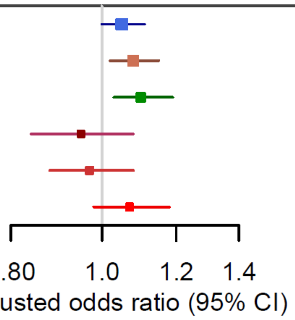

Figure S1: Odds of stunting a 1-log higher median fecal indicator bacteria concentrations in drinking water, on child hands, on fomites, in soil, and food, and a 1-log higher in median kitchen fly density.

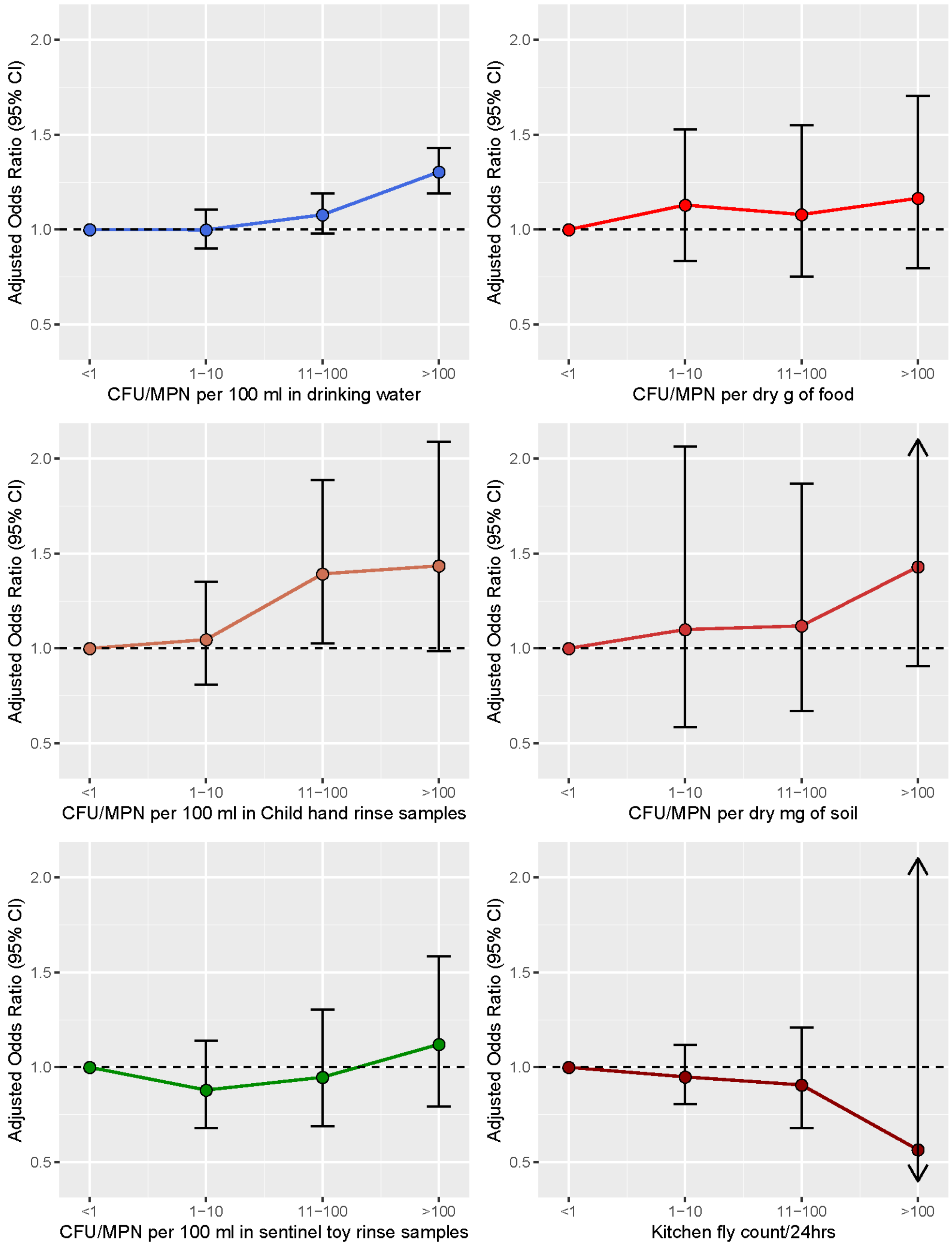

Figure S2: Odds of diarrhea for 1-log higher in fecal indicator bacteria concentrations in drinking water, child hands, fomites, food, soil and 1-log higher in kitchen fly density, stratified by log category.

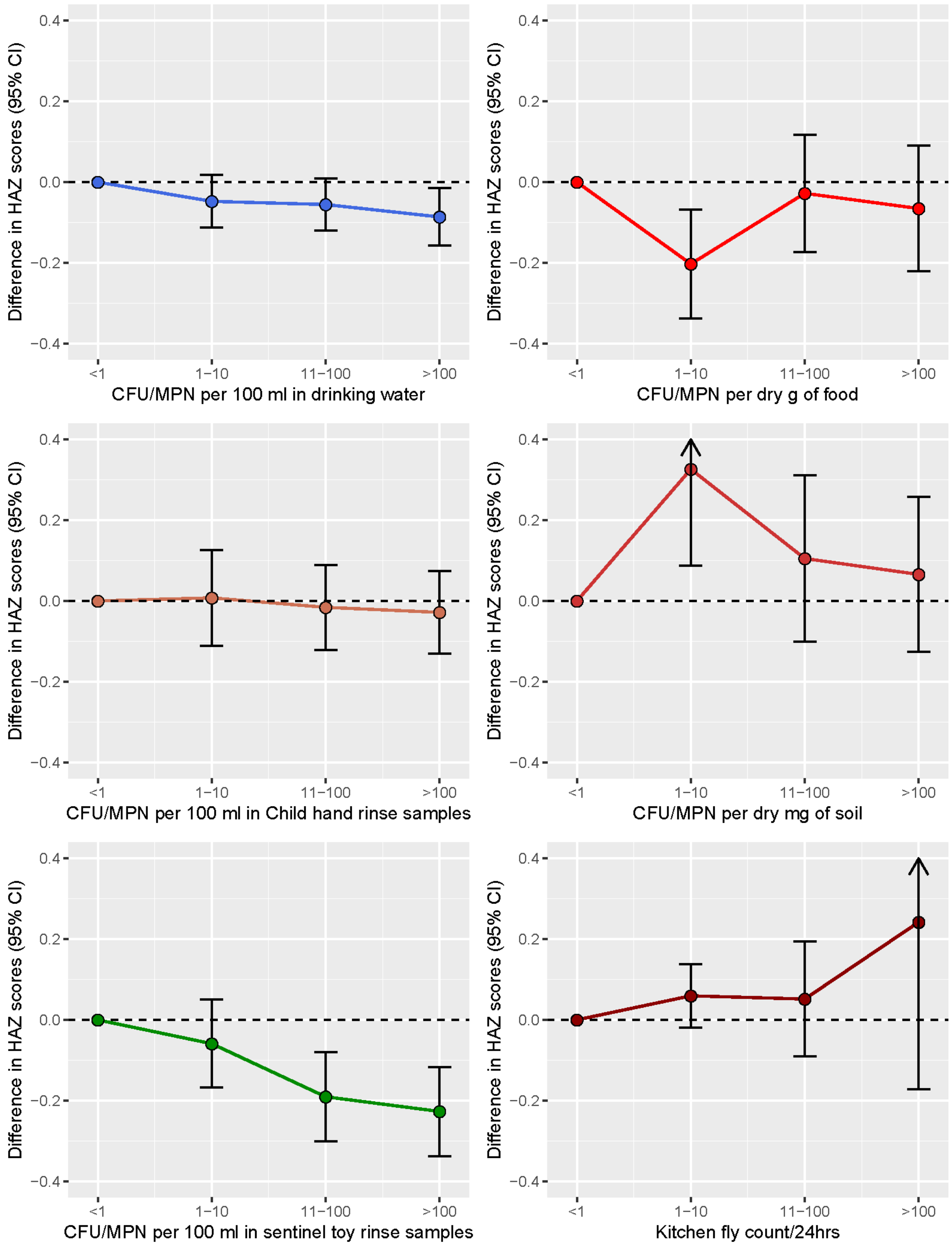

Figure S3: Difference in height-for-age Z score for 1-log higher fecal indicator bacteria concentrations in drinking water, child hands, fomites, food, soil and a 1-log higher kitchen fly density, stratified by log category

.

Pathway	Studies	N	OR (95% CI)	I2 (95% CI)
Drinking water	19	43,337	1.08 (1.04, 1.12)	26% (0-58%)
Child hands	4	5,387	1.11 (1.02, 1.22)	0% (0-69%)
Fomites	2	2,735	1.05 (0.95, 1.16)	0% (-)
Fly density	4	6,548	0.95 (0.82, 1.11)	10% (0-86%)
Soil	1	2,376	1.11 (0.99, 1.24)	-
Food	1	2,166	1.03 (0.91, 1.17)	-

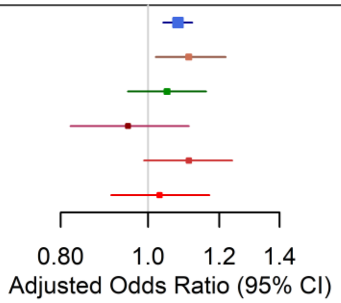

Figure S4: Odds of diarrhea a 1-log higher fecal indicator bacteria concentrations in drinking water, on child hands, on fomites, in soil, and food, and a 1-log higher in kitchen fly density, using six log_{10} categories (<1, 1-10, 11-100, 101-1,000, 1,001-10,000, 10,000+) instead of four as the explanatory variable.

Pathway	Studies	N	OR (95% CI)	I2 (95% CI)
Drinking water	19	43,337	1.09 (1.05, 1.13)	18% (0-53%)
Child hands	4	5,387	1.11 (1.02, 1.21)	2% (0-85%)
Fomites	2	2,735	1.09 (0.98, 1.20)	0% (-)
Fly density	4	6,548	1.02 (0.89, 1.17)	0% (0-85%)
Soil	1	2,376	1.15 (1.02, 1.31)	-
Food	1	2,166	1.05 (0.94, 1.17)	-

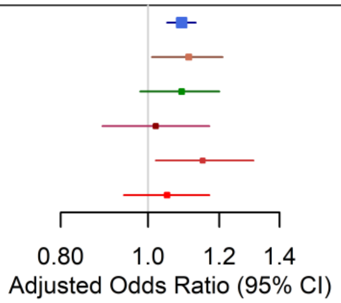

Figure S5: Odds of diarrhea a 1-log higher fecal indicator bacteria concentrations in drinking water, on child hands, on fomites, in soil, and food, and a 1-log higher kitchen fly density, using a continuous log_{10} transformation as the explanatory variable instead of log_{10} categories.

Pathway	Studies	N	HAZ (95% CI)	I2 (95% CI)
Drinking water	7	10,888	-0.04 (-0.06, -0.01)	23% (0-66%)
Child hands	3	5,345	-0.04 (-0.11, 0.04)	67% (0-90%)
Fomites	2	3,178	-0.05 (-0.08, -0.02)	0% (-)
Fly density	3	3,966	0.04 (-0.02, 0.11)	0% (0-88%)
Soil	1	1,723	-0.02 (-0.06, 0.03)	-
Food	1	1,586	-0.03 (-0.07, 0.02)	-

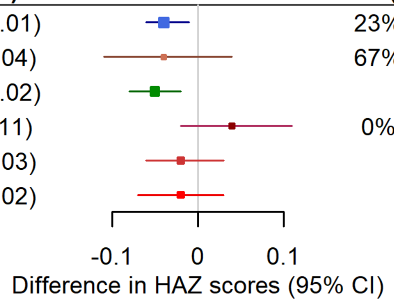

Figure S6: Difference in height-for-age Z score for 1-log higher median fecal indicator bacteria concentrations in drinking water, on child hands, on fomites, in soil, and food, and 1-log higher median kitchen fly density, using six log_{10} categories (<1, 1-10, 11-100, 101-1,000, 1,001-10,000, 10,000+) instead of four as the explanatory variable.

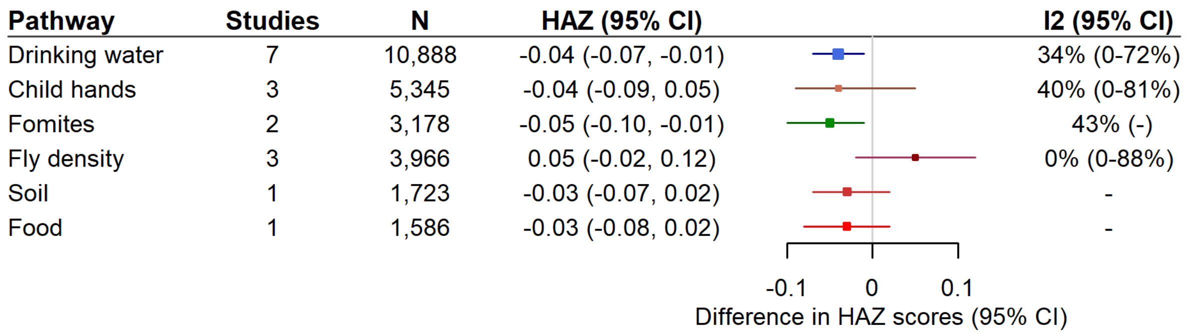

Figure S7: Difference in height-for-age Z score for 1-log higher median fecal indicator bacteria concentrations in drinking water, on child hands, on fomites, in soil, and food, and 1-log higher median kitchen fly density, using a continuous $\log_{10}$ transformation as the explanatory variable instead of $\log_{10}$ categories

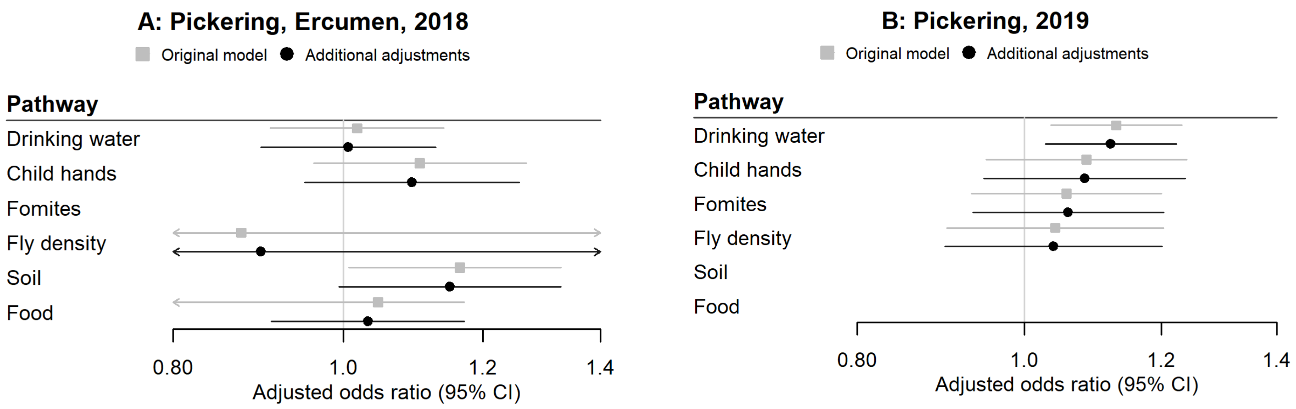

Figure S8: Odds of diarrhea for 1-log10 higher fecal indicator bacteria concentrations in drinking water, child hands, fomites, food, soil and 1-log10 higher kitchen fly density, comparing findings from the original models to those that include additional covariates in the WASH-Benefits Bangladesh (A) and Kenya (B) data.

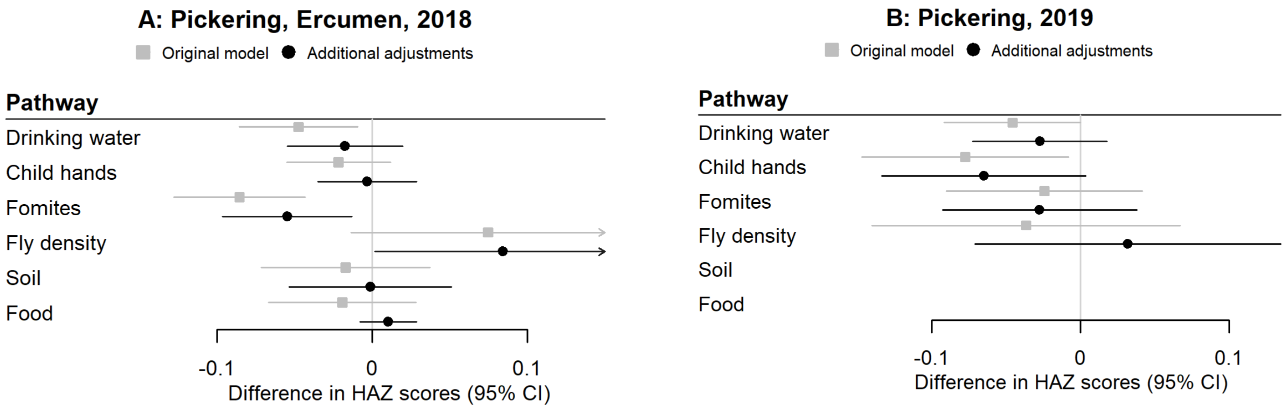

Figure S9: Difference in height-for-age Z scores for 1-log10 higher fecal indicator bacteria concentrations in drinking water, child hands, fomites, food, soil and 1-log10 higher kitchen fly density, comparing findings from the original models to those that include additional covariates in the WASH-Benefits Bangladesh (A) and Kenya (B) data.

Appendix C – Risk of Bias Assessments

Diarrhea

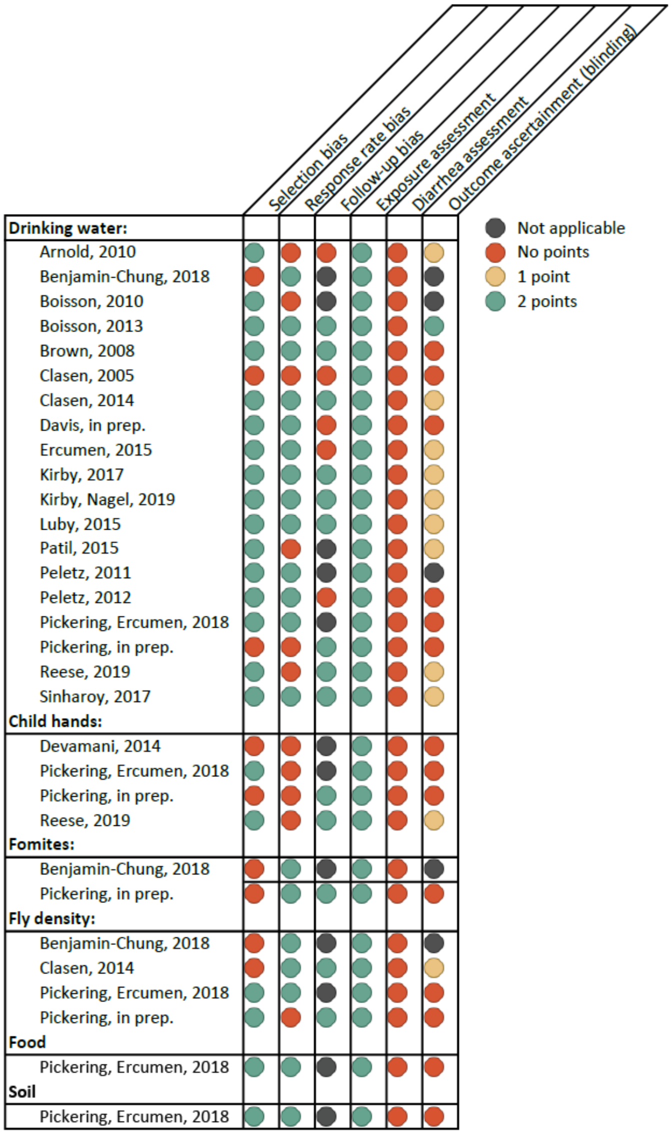

Figure S10: Risk of bias assessment for studies included in the diarrhea analyses

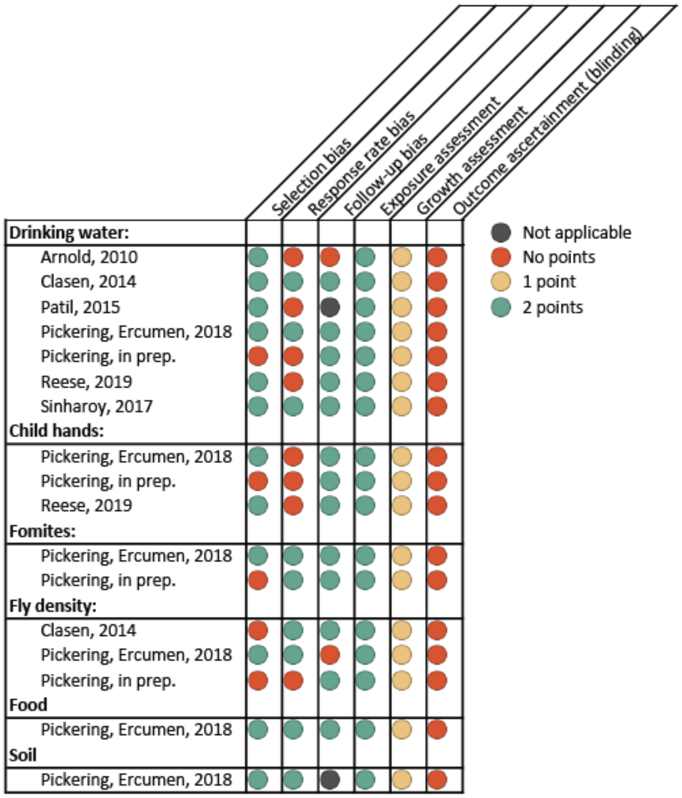

Figure S11: Risk of bias assessment for studies included in the growth analyses
